# Methodological challenges in the genomic analysis of an endangered mammal population with low genetic diversity

**DOI:** 10.1038/s41598-022-25619-y

**Published:** 2022-12-10

**Authors:** Lídia Escoda, Oliver Hawlitschek, Jorge González-Esteban, Jose Castresana

**Affiliations:** 1 Institute of Evolutionary Biology (CSIC-Universitat Pompeu Fabra), Passeig Marítim de la Barceloneta 37, 08003 Barcelona, Spain; 2 Leibniz Institute for the Analysis of Biodiversity Change, Centre for Molecular Biodiversity Research, Zoological Museum, Martin-Luther-King-Platz 3, 20146 Hamburg, Germany; 3 Desma Estudios Ambientales S.L., Sunbilla, Navarra Spain

**Keywords:** Conservation genomics, Genetic variation

## Abstract

Recently, populations of various species with very low genetic diversity have been discovered. Some of these persist in the long term, but others could face extinction due to accelerated loss of fitness. In this work, we characterize 45 individuals of one of these populations, belonging to the Iberian desman (*Galemys pyrenaicus*). For this, we used the ddRADseq technique, which generated 1421 SNPs. The heterozygosity values of the analyzed individuals were among the lowest recorded for mammals, ranging from 26 to 91 SNPs/Mb. Furthermore, the individuals from one of the localities, highly isolated due to strong barriers, presented extremely high inbreeding coefficients, with values above 0.7. Under this scenario of low genetic diversity and elevated inbreeding levels, some individuals appeared to be almost genetically identical. We used different methods and simulations to determine if genetic identification and parentage analysis were possible in this population. Only one of the methods, which does not assume population homogeneity, was able to identify all individuals correctly. Therefore, genetically impoverished populations pose a great methodological challenge for their genetic study. However, these populations are of primary scientific and conservation interest, so it is essential to characterize them genetically and improve genomic methodologies for their research.

## Introduction

Some of the main threats that can affect the viability of a population include habitat loss and fragmentation, which, often, lead to the isolation of small populations and, consequently, reduced genetic variation and inbreeding^1,2^. Loss of genetic diversity may have detrimental effects on both population fitness and viability in the long term by restricting evolutionary potential^3^. In addition, inbreeding, caused by mating between close relatives, can lead to inbreeding depression, reducing fitness and population growth in the short term^4^. Both of these factors significantly increase the probability of extinction of populations.

Indeed, a growing number of studies based on complete genomes have recently revealed very low genetic diversity in certain mammalian species of conservation concern. In some of these, heterozygosity is as low as ~ 100 SNPs/Mb (heterozygous sites or SNPs per mega-base), one order of magnitude below that of species with large populations. These include species such as the vaquita (*Phocoena sinus*), with 105 SNPs/Mb^5^, and the Iberian lynx (*Lynx pardinus*), with 102 SNPs/Mb^6^. In a few other species, the heterozygosity is yet another order of magnitude smaller, such as in the Island fox (*Urocyon littoralis*), with just 14 SNPs/Mb found in an individual from San Nicolas Island^7^. How these species have reached these extraordinarily low levels of genetic diversity and how this may be aggravated by consanguinity and inbreeding depression is still unclear^2,8–11^. To address these questions, genomic analyses of populations with extremely low genetic diversity are essential.

The Iberian desman (*Galemys pyrenaicus*), also known as the Pyrenean desman, is a small semi-aquatic mammal that inhabits clean rivers in mountains of the northern half of the Iberian Peninsula^12,13^. In recent years, the species has suffered significant population declines for reasons that are still being analyzed, but loss and fragmentation of the riparian habitat appears to be one of the most critical factors^14^. Previous double digest restriction site-associated DNA (ddRAD) and whole-genome sequencing studies of the species revealed that the Iberian desman has exceptionally low heterozygosity levels^15,16^. The lowest levels were recorded in the Pyrenees, where heterozygosity ranged from 12 to 116 SNPs/Mb, covering the two lowest orders of magnitude reported in mammals. Studies based on kinship networks revealed important connectivity problems for the species due to physical barriers, such as dams, which are leading to populations with very high inbreeding levels in the upper parts of rivers^17,18^. Ecological barriers, including the desiccation of rivers, the presence of predators such as the American mink, and contamination coming from urban areas^14^, can also isolate populations.

Here, we analyze a population of Iberian desman from the western Pyrenees, where it was previously shown, using relatively few individuals, that the species exhibits shallow genetic diversity throughout the area^15,16^. To obtain genomic sequences and SNPs for the analyses, we applied the ddRADseq (ddRAD sequencing) technique, which is popular in population genomic studies due to the ease of obtaining data from a large number of individuals^19^, and assembled the reads using the recently sequenced Iberian desman genome^15^. We determined the heterozygosity rate and the inbreeding coefficient for 45 individuals to describe the genetic diversity of this population in detail and to understand whether genetic diversity and inbreeding were homogeneous in this area or whether these values were more extreme in some particular localities. We also tested the resolution power of the SNPs obtained by ddRADseq to genotype individuals in these populations of extremely low genetic variability and showed that most of the methods tested were unable to perform a correct individualization. Solving these methodological problems is crucial to address critical conservation problems in populations of species with extremely low genetic diversity.

## Materials and methods

### Samples of the Pyrenean desman

**Figure 1:**
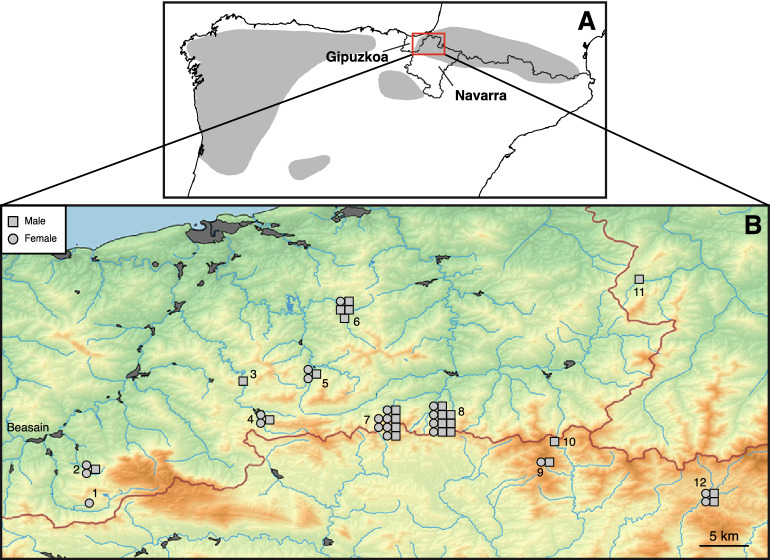
(**A**) Map of the Iberian Peninsula showing the Iberian desman distribution range in grey. The study area is indicated with a red square. (**B**) Enlarged map showing the localities of the Iberian desmans used in this work. Each sampled individual is represented with different symbols for males (squares) and females (circles). The numbers indicate the localities (1: Aiaiturrieta-Ataun; 2: Amundarain-Zaldibia; 3: Leitzaran-Berastegi; 4: Erasote-Leitza; 5: Urumea; 6: Elama-Artikutza; 7: Ameztia-Labaien; 8: Ezpelura-Urrotz; 9: Sasoaran-Eugi; 10: Olazar-Eugi; 11: Aritzakun; and 12: Urrobi-Auritz). Red lines delimit the three main river basins: Cantabrian at the northwest, Adur at the northeast, and Ebro at the south. Populations 1 and 2 are possibly extinct. Human population nuclei with more than 500 inhabitants are indicated in dark gray. The map was constructed using QGIS 2.14^53^. The distribution range of *Galemys pyrenaicus* was generated using information from different sources^13,54^. The coastline shapefile was downloaded from Natural Earth (https://www.naturalearthdata.com). The shapefiles of the shaded relief map, rivers, main river basins, and human population nuclei were downloaded from Instituto Geográfico Nacional (https://www.ign.es). The river shapefile for France was obtained from DIVA-GIS (https://www.diva-gis.org).

The population studied is located in the provinces of Gipuzkoa and Navarra (Spain), in the north of the Iberian Peninsula (Fig. 1). We used 45 tissue samples from Iberian desmans (a small piece from the tail tip) captured between 1997 and 2011 during monitoring works of the species promoted by the environmental authorities Diputación Foral de Gipuzkoa and Gobierno de Navarra (Supplementary Table S1 of the Supporting Information and Fig. 1). From these, 13 samples had already been used in previous works^15,16,20^.

### Ethics statement

No animal was specifically captured for this study, as they had been captured for previous works to monitor these populations. Therefore, this study did not require ethics approval by a specific committee.

### Construction of ddRAD libraries and sequence processing

DNA was extracted using the DNEasy Blood and Tissue Kit (QIAGEN). Genomic libraries were constructed following the ddRADseq protocol^19^ with some modifications. Each library was made in groups of 24 samples, and samples with low sequence yield were repeated in subsequent experiments, as indicated in Supplementary Table S1. First, the DNA was digested using the restriction enzymes EcoRI and MspI. The digested DNA was ligated to adapters P1 and P2, which bind to the EcoRI and MspI overhangs, respectively. Adapter P1 contains a different 5-nucleotide barcode for each sample so that they can be identified in the library sequences. All samples were then pooled and a fraction between 300 and 400 bp was selected in an E-Gel EX 2% agarose gel (Invitrogen). From this fraction, 16-cycle PCR amplifications were carried out using primers that anneal to the adapters and allow the generation of standard Illumina libraries. To minimize sequencing bias, 6 PCRs were performed for each sample. PCR products were concentrated in 20 μl using the MinElute PCR Purification Kit (QIAGEN). Finally, the library concentration was estimated using a Nanodrop, 400 ng were run in an EX 2% agarose gel, and the library was extracted in 30 μl using the QIAquick Gel Extraction Kit (QIAGEN). The libraries were sequenced using the NextSeq Sequencing System (Illumina) in the Genomics Core Facility at Pompeu Fabra University with the 150-cycle Mid Output kit and single-read sequencing.

The sequences obtained were filtered and assembled using the Stacks 2.60 package^21^. First, the process_radtags program was used to separate reads belonging to different individuals according to the barcodes using the *recovery* option (-r) and to filter out reads with a quality score limit (-s) of 10. After this step, reads from samples sequenced in different libraries were combined. This set of filtered reads is available at Dryad (see Data Availability section). Then, the reads were mapped to the reference genome of the Iberian desman^15^ with BWA v0.7.17^22^ using the *mem* algorithm. Subsequently, SAMtools v1.9^23^ was used to produce BAM alignments where reads with a minimum mapping quality (-q) of 20 were kept. The mapped reads were processed using Gstacks from the Stacks package with the SNP calling model *snp* and both alpha thresholds for discovering SNPs and for calling genotypes of 0.01. Using the populations program of the same package, the sequences were saved in FASTA format after selecting loci with a minimum proportion of called individuals (r) of 0.9 (i.e., loci present in at least 90% of the individuals) and a minor allele frequency (MAF) of 0. Using the FASTA sequences of the loci, heterozygosity was estimated for each specimen as the number of heterozygous positions divided by the total sequenced length of the loci. The SNP dataset of all samples containing the first SNP from each locus was obtained with r = 0.9 and MAF = 0.025 and it was filtered for linkage disequilibrium (r^2^ > 0.8) with PLINK v1.90b6.22^24^. These SNPs were saved in PLINK and VCF formats for further analyses.

The sex of all individuals was determined bioinformatically by mapping the reads against a Y-chromosome loci database as in previous work^16^.

### Population structure analysis

Population structure was analyzed with STRUCTURE 2.3.4^25^ using the admixture model, correlated allele frequencies, and a number of populations (K) ranging from 1 to 6. A total of 1,000,000 iterations were run with a burn-in of 100,000. For each K value, 10 independent runs were performed and summarized with CLUMPP^26^. The optimal value of K was estimated with the method of Evanno et al.^27^, as implemented in STRUCTURE HARVESTER^28^.

The principal component analysis (PCA) was performed with the KING v.2.2.5 program^29^.

### Relatedness and inbreeding coefficients

We calculated the kinship coefficients between pairs of Iberian desmans with the KING program^29^. KING uses a method to infer the kinship coefficient between pairs of individuals which does not require allele frequency information. Specifically, we used the KING-robust method, which is not affected by population structure, with the *kinship* option. Negative kinship values and pairs with a flag error of 0 or 0.5, corresponding to unrelated individuals, were not used. The kinship coefficients were doubled to convert them to relatedness coefficients.

The relatedness coefficients were also calculated using the RELATED program^30,31^. We used the *dyadml* estimator^32^, which was previously shown to be the most adequate for SNPs derived from ddRADseq data^18^, and the full nine states identity-by-descent (IBD) model, which takes inbreeding into account. Confidence intervals (95%) were calculated with 100 bootstraps, and only relatedness values with confidence intervals that did not overlap 0 were used. Using the same model, we estimated the inbreeding coefficient for each Iberian desman. The inbreeding coefficient was also estimated with PLINK^24^, which estimates this value based on the observed versus expected number of homozygous genotypes. It should be taken into account that these two estimates of the inbreeding coefficient, based on SNP frequencies without genomic position information, measure relatively recent inbreeding events and do not reflect past inbreeding, as do measures based on runs of homozygosity estimated from whole genomes^15^.

### Test of individualization using duplicate samples

As the relatedness coefficients were obtained from specimens with very low genetic diversity and very high inbreeding levels, we tested the accuracy of the estimates in this special context. For this, we used the reads of duplicate samples from the same individual that were repeated in different runs to increase their coverage (Supplementary Table S1). Only samples with a minimum of 200,000 reads, which were found to be sufficient for this purpose in preliminary analysis, were compared in a new assembly. In it, duplicate samples were included, without combining them, together with all the other samples in the study, giving rise to the comparison of 23 replicated samples. In addition, we recorded how these programs behaved when comparing three individuals from Amundarain-Zaldibia, with extreme inbreeding levels and which were probably related to one another. We had two samples for one of these individuals, leading to five comparisons between highly inbred and related individuals. For this analysis, we generated the SNP dataset with the same parameters as above. In addition, we generated three extra datasets with higher MAF filter values (0.1, 0.2, and 0.3, respectively) to test the effect of this parameter.

The samples were individually identified using different methods. The KING program and the RELATED program with the three-states IBD model (with no inbreeding) were used to estimate relatedness coefficients and the individualization was performed considering that duplicate samples have a relatedness coefficient of 1^33^. We also used the *duplicate* option in KING to detect duplicates automatically. Finally, we tested other programs for detecting duplicates and kinship relationships: the clustering method implemented in PLINK using the "*–cluster –matrix*" option^24^; COLONY v2.0.6.5 with allelic dropout and false allele rates of 0.001 each^33,34^, as they proved to be the best error rates in preliminary analyses; and the pedigree-reconstruction methods PRIMUS v1.9.0^35^ and VCF2LR^36^.

### Test of relatedness, individualization and inbreeding estimates using simulated pedigrees

We also tested the accuracy of relatedness estimates and individualization using simulations of three artificial pedigrees, in which genotypes from our dataset were bioinformatically crossed using the script GetCrosses^18^. Each pedigree had founders from different areas (Supplementary Fig. S1). We also assessed the accuracy of the inbreeding coefficient calculations from RELATED using three pedigrees with additional crosses between relatives (Supplementary Fig. S2). In both cases, individuals with exceptional inbreeding levels were excluded as founders, leaving founders with inbreeding values that ranged from 0.0175 to 0.4783, to produce more generally applicable results. A total of 100 simulations were performed for each pedigree.

## Results

### Sequence assembly

After mapping the reads for each of the 45 Iberian desman to the reference genome (Supplementary Table S2), a total of 43,478 loci were assembled and a set of 1421 SNPs present in at least 90% of the individuals was generated. The genetic sexing led to the determination of 21 females and 24 males (Supplementary Table S1).

### Population structure

**Figure 2:**
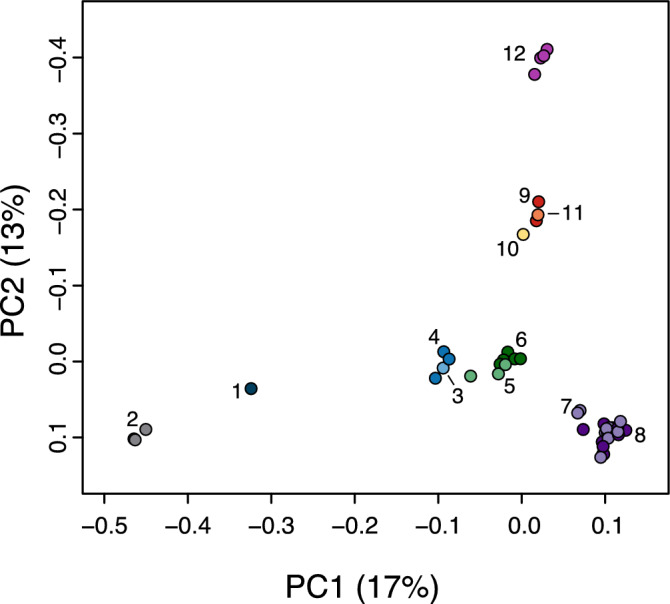
Principal component analysis of the Iberian desman individuals. Each locality is represented with a different color. The locality code, which can be found in the legend of Fig. 1, is also indicated.

Both the PCA (Fig. 2) and the STRUCTURE (Supplementary Fig. S3) analyses showed a certain degree of structure in the area. The optimal value of populations in STRUCTURE was K = 2, followed by a secondary peak at K = 4 (Supplementary Fig. S3B).

### Heterozygosity and inbreeding coefficients

Heterozygosity rates were very low for all the individuals, ranging from 26 to 91 SNPs/Mb (Supplementary Table S3 and Supplementary Fig. S4). As ddRADseq data is a subset of the genome, heterozygosity values calculated here may not be identical to those obtained from the whole genome sequence, but they are within the same order of magnitude. For example, for one desman from which the whole genome was obtained (IBE-C2769), heterozygosity was 116 SNPs/Mb when calculated from the whole genome^15^ and 80 SNPs/Mb from the ddRADseq data (Supplementary Table S3).

**Figure 3:**
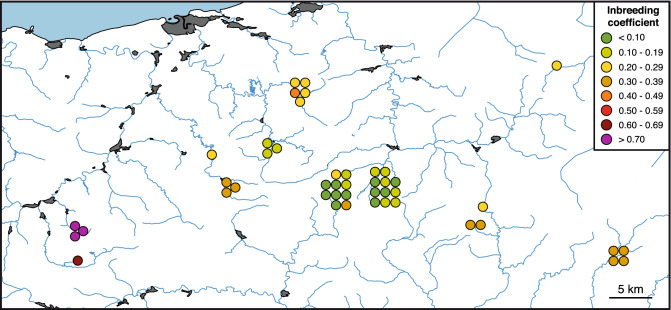
Map of the Iberian desmans with their inbreeding coefficients, estimated with RELATED, represented using a color scale. The map was constructed as indicated in the legend to Fig. 1.

Individual inbreeding coefficients estimated with RELATED showed a mean value of 0.25 (Supplementary Table S3) and were highly variable among localities. The map showing color-coded values (Fig. 3) revealed exceptional inbreeding coefficients in the two most occidental localities, Amundarain-Zaldibia and Aiaiturrieta-Ataun, where most coefficients were higher than 0.7 and the four individuals presented a mean value of 0.76. In contrast, in two central localities within the analyzed area, Ezpelura-Urrotz and Ameztia-Labaien, the individuals had the lowest inbreeding values, with a mean of 0.11. Heterozygosity and inbreeding coefficient showed a strong negative correlation (R = − 0.93) when considering just this population of the Iberian desman.

Individual inbreeding coefficients estimated with PLINK showed similar results, with a mean value of also 0.25 (Supplementary Table S3).

### Pairwise relatedness and connectivity networks

**Figure 4:**
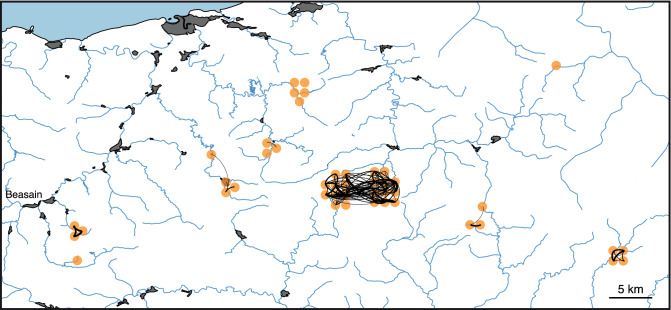
Maps plotting networks of related individuals detected by KING. Line thickness is proportional to the relatedness coefficient of the connected Iberian desmans. The map was constructed as indicated in the legend to Fig. 1.

We found 114 relationships between pairs of Iberian desmans determined using KING and 382 with RELATED. To visualize the connectivity patterns in the area, we represented the kinship networks on a map (Fig. 4 and Supplementary Fig. S5 for KING and RELATED relationships, respectively). As the relationships derived from RELATED were more abundant, these were subdivided into close and distant relationships according to a 0.2 threshold. Both relationships from KING and close relationship from RELATED predominantly showed connectivity between individuals from the same river or from neighboring localities. Only distant relationships determined using RELATED (Supplementary Fig. S5B) showed connectivity between individuals from different basins.

### Evaluation of the power to perform individualization and relatedness

**Figure 5:**
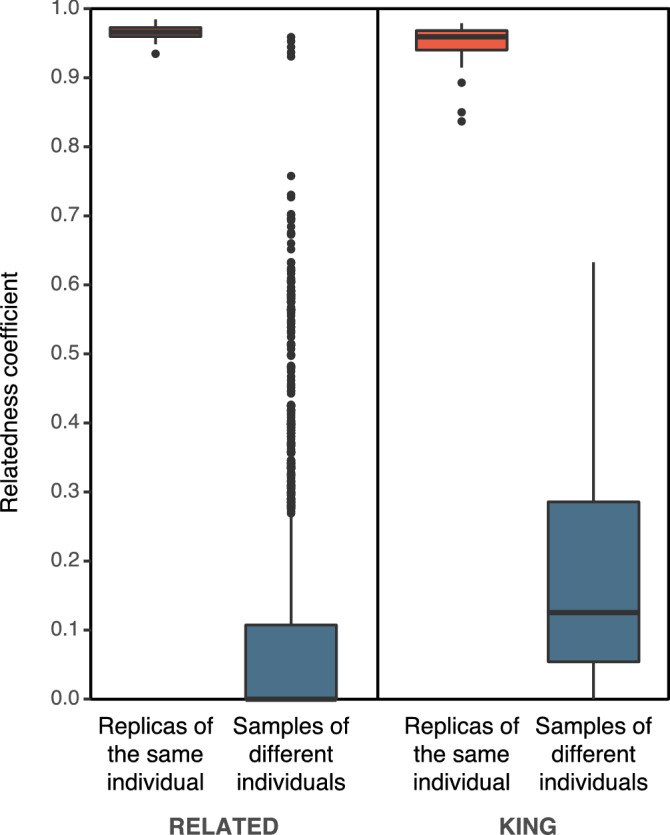
Boxplots of relatedness coefficients obtained using RELATED and KING for the analysis of replicates of the same individual and samples of different individuals.

We first tested the accuracy of the individualization by including 23 known pairs of replicates from different ddRAD libraries in a new assembly that rendered 688 SNPs. Theoretically, the relatedness coefficient between samples of the same individual should be 1. Both RELATED and KING showed values of this parameter close to 1 for all the pairs of replicates (Fig. 5; > 0.9 for RELATED and > 0.8 for KING), which, in principle, would be a good result in most situations as these values are much higher than the highest theoretical relatedness value between different individuals (0.5 for first-degree relationships). Thus, when applying a threshold of 0.8, which is the most favorable for both programs, all 23 replicates were detected (Table 1). As for the other options and programs used, the *duplicate* option from KING was able to detect only 21 out of 23 pairs of replicates whereas PLINK, COLONY, PRIMUS, and VCF2LR detected all the duplicate pairs.

**Table 1:** Results of the individual identification performed with different programs. Correct detection of replicas corresponds to the detection of the 23 pairs of duplicated samples. Correct detection of highly inbred individuals corresponds to the detection of the 4 available sequencing experiments of the 3 individuals from Amundarain-Zaldibia.

Program	Correct detection of replicas	Correct detection of highly inbred individuals
RELATED	23/23	0/5
KING	23/23	5/5
KING -*duplicate*	21/23	5/5
PLINK	23/23	0/5
COLONY	23/23	0/5
PRIMUS	23/23	0/5
VCF2LR	23/23	0/5

Distinguishing samples of different individuals is more problematic under high inbreeding scenarios because the relatedness values are much greater than the theoretical ones. When considering samples of different individuals, we evaluated whether these programs could discern three closely related individuals with very high inbreeding levels from Amundarain-Zaldibia. For one of these we had two samples, enabling five comparisons of related and highly inbred individuals. Indeed, RELATED showed relatedness values > 0.9 between samples known to belong to these different individuals, overlapping with the values found for replicate samples and therefore incorrectly individualizing the inbred desmans (Fig. 5 and Table 1). In the case of KING, all estimations between the different individuals, including the most inbred, were lower than 0.8, and thus lower than all the values between replicate samples, meaning that it correctly distinguished the inbred individuals (Fig. 5 and Table 1). PLINK, COLONY, PRIMUS, and VCF2LR also considered the highly inbred specimens to be replicates, giving erroneous individualization results (Table 1). Similar results for the identification of replicated and different individuals were obtained for all programs when different MAF filters were used (Supplementary Table S4), so it is probably better to use a MAF filter that does not remove too many SNPs.

We also tested the reliability of the relatedness coefficients estimated by simulating artificial pedigrees (Supplementary Fig. S6). We found a general overestimation of all the relationships tested in the three pedigrees, but the relatedness coefficients obtained using RELATED (Supplementary Table S5) were, in most cases, higher than those estimated with KING (Supplementary Table S6). Overestimation increased as more distant relationships were simulated. Only in the cases of parent–offspring and grandparent-grandchild, KING underestimated the relatedness coefficient (Table 2).

**Table 2:** Average relatedness values and standard deviations (in parentheses) estimated with different simulated pedigrees using the programs RELATED and KING in comparison with the expected values. The values corresponding to each pedigree can be found in Supplementary Tables S4 and S5.

Relationship	Theoretical value	Observed value	
		RELATED	KING
Parent-offspring	0.5	0.6086 (0.0597)	0.4636 (0.0379)
Full-siblings	0.5	0.6080 (0.0234)	0.5699 (0.0280)
Half-siblings	0.25	0.3220 (0.0242)	0.2840 (0.0329)
Grandparent-grandchild	0.25	0.3304 (0.0348)	0.1475 (0.0792)
Uncle-nephew	0.25	0.3237 (0.0295)	0.2861 (0.0396)
Half uncle-half nephew	0.125	0.2692 (0.0421)	0.2335 (0.0524)
Half-first cousins	0.0625	0.2243 (0.0289)	0.1822 (0.0431)

To test the performance of the inbreeding coefficient estimates with RELATED, we simulated different pedigrees with inbreeding (Supplementary Fig. S7). We found highly accurate estimates of the inbreeding coefficients in both the western and central areas, and only in pedigrees from the eastern area were some of the values obtained much higher than expected (Table 3 and Supplementary Table S7).

**Table 3:** Average individual inbreeding coefficients and standard deviations (in parentheses) estimated with different simulated pedigrees of offspring (in parentheses) from different types of parental relationships in comparison with the expected values. Offspring codes can be found in Supplementary Fig. S2. The values corresponding to each pedigree can be found in Supplementary Table S6.

Parental relationship (offspring)	Theoretical value	Observed value
None (F1, F201, F202)	0	0.0099 (0.0141)
Full-siblings (F203, F205)	0.25	0.2762 (0.0311)
Half-siblings (F204)	0.125	0.1264 (0.0325)
Half-first cousins (F301)	0.03125	0.0811 (0.0295)

## Discussion

### Genetic analyses in populations with extremely low genetic diversity using ddRADseq

Identification of individuals from genetic marker data is important in many studies involving the monitoring of elusive species, for example, in estimating population density with capture-recapture, either using trapped specimens or non-invasive samples^37,38^. When there is ample marker information, individual identification is straightforward using various methods, including mismatch methods, pairwise relatedness analysis, or the COLONY program to handle multiple replicates^33,39,40^. Nevertheless, few studies have addressed the issue of differentiating them under conditions of both low genetic diversity and high levels of inbreeding, where individuals share a large proportion of their genotype^41^. In particular, exceptionally high inbreeding and low heterozygosity levels mean that some individuals appear to be almost clones at the genomic level. This is the case with the three highly inbred desman individuals from Amundarain-Zaldibia, which have an average inbreeding coefficient of 0.78 and an average heterozygosity of 27.3 SNPs/Mb, as well as a high degree of kinship between them, making it very difficult for the available individual identification programs to distinguish between replicates of the same individual and samples of different individuals (Fig. 5 and Table 1). In this study, we were able to use tissue samples of known origin and replicate libraries, making it a unique opportunity to address the methodological problems for detecting individuals in a context of low genetic diversity and high inbreeding levels. Of all the programs tested, those based on the pairwise relatedness between individuals (RELATED and KING) gave the best individualization results, as already shown in other studies^39^. In particular, KING is the only program that was able to differentiate between replicates of the same individual and samples of different individuals with high inbreeding levels (Table 1). The RELATED program was able to allocate all the known replicates, but failed to distinguish the highly inbred individuals as different samples. The different performance between RELATED and KING is probably due to the fact that the likelihood estimator implemented in RELATED uses the allele frequencies of the given set of samples to calculate the relatedness coefficient^32^, while the KING algorithm uses only SNP data from the pair of individuals tested each time, which makes the inference robust to the presence of population structure^29^. The main problem with algorithms that use allele frequencies, such as RELATED, is that they assume a homogeneous population structure and lead to inflated results among individuals of the same group when this condition is not fulfilled^29^; this is likely to happen in populations with certain population structure (Fig. 2 and Supplementary Fig. S3) and low levels of connectivity (Fig. 4 and Supplementary Fig. S5), such as those in this study.

This difference between programs in the power to estimate the relatedness coefficient can also be observed in the simulations with artificial pedigrees, where the estimates produced using RELATED were systematically inflated for most of the tested kinship categories compared to those obtained with KING (Table 2 and Supplementary Fig. S6). In any case, the simulations proved that, despite not being able to categorize the specific kinship categories, it is possible to detect a certain level of relatedness between the samples analyzed and construct relatedness networks to assess the level of connectivity in the area.

Estimations of the inbreeding coefficient of the artificial pedigrees using RELATED were generally more accurate than the relatedness coefficient (Table 3 and Supplementary Fig. S7), as observed in previous simulation works of populations with a higher genetic diversity and lower inbreeding^17,18^. This indicates that inbreeding estimations are more robust to the presence of population stratification and work well with both low and high levels of inbreeding. This is an important result because it allows the assessment of genetic health in inbred and low-diversity populations. The fact that the PLINK program, whose estimates are based on a different principle, gives inbreeding coefficients very similar to those obtained by RELATED, is another point that gives confidence to these results.

### Extremely low genetic diversity and high inbreeding levels in the Iberian desman

Heterozygosity was unusually low in all specimens (Supplementary Table S3), with values ranging from 26 to 91 SNPs/Mb. These values are among the lowest found for the Iberian desman across its entire range^15,16^. In fact, they are among the smallest found for any mammal so far, only comparable to those found for the endangered and completely isolated Channel Island fox^7^, as estimated from its whole genome. It should be noted that heterozygosity values estimated from ddRADseq are not exactly the same as those estimated from whole genome data, but they are within the same order of magnitude^15^. Many other species have proven to prevail for long periods of time with low levels of genetic diversity^5–7,42–44^. Low genetic diversity diminishes the evolutionary potential of populations^3^, making it difficult to understand how these populations are able to survive with these exceedingly low levels of genetic diversity. Recent work based on genomic data suggests that these populations may have a low mutational load (i.e., a small proportion of deleterious recessive mutations that could become homozygous under inbreeding), as a consequence of purging of recessive strongly deleterious mutations in the past^9,45,46^. However, inbreeding should also be taken into account in these populations because it can have a negative impact when weakly and mildly deleterious variants, very difficult to purge, become homozygous^4,47^. Consequently, inbreeding may be a critical factor for predicting the fate of these low-diversity populations.

Indeed, the inbreeding coefficients determined for the Iberian desmans in this area were of great interest as they varied widely among the different localities (Fig. 3). In the central populations, where the density of the species is higher, inbreeding was relatively low, with many individuals having values < 0.1 and thus of an acceptable level for wild animals^3^. In contrast, there are some populations with extremely high values. In particular, the four individuals from Amundarain-Zaldibia and Aiaiturrieta-Ataun exhibited extremely high levels of inbreeding (Fig. 3, Supplementary Table S3). These values are typical of critically endangered species or populations, including the Attwater’s prairie-chicken (*Tympanuchus cupido attwateri*), with values of 0.65 in some individuals^48^; and grey wolves (*Canis lupus*) of the highly inbred population on Isle Royale, with values of 0.81^49^. The presence of highly industrialized areas and large human population nuclei, such as Ordizia and Beasain, downstream from the Amundarain-Zaldibia and Aiaiturrieta-Ataun desman populations, could have played a role in isolating these individuals from the other populations. Specifically, the channeling of the rivers and the destruction of the riparian habitat, together with water pollution around these urban areas, could create ecological barriers to dispersal and favor the inbreeding of the isolated populations. No close relationships were found between individuals from this area and the rest (Fig. 4 and Supplementary Fig. S5), so each population may have been isolated for several generations. Probably as a consequence of this, no desman was recorded in Aiaiturrieta-Ataun after 2001 and in Amundarain-Zaldibia after 2006 (the latest surveys having been carried out in 2018), suggesting that these populations could be extinct and are likely an example of the inbreeding-driven extinction vortex^50–52^. Early knowledge of this problem and an action aimed at promoting dispersal from genetically healthier nearby populations to these strongly inbred populations, whether natural or assisted, could have helped to reverse this situation.

There are other isolated desman populations of the Iberian desman with very low genetic diversity that could follow a similar extinction trajectory. Studies should be promoted to characterize not only their demographic status, but also the heterozygosity and inbreeding levels of these populations, in order to evaluate their conservation status and take the necessary measures to ensure their long-term viability.

## Supplementary Information

Supplementary Information.

## Data Availability

Filtered ddRADseq data is available in Dryad (https://doi.org/10.5061/dryad.brv15dvck). Additional data and figures may be found in Supporting Information.
