# Supplementary material for: Methodological challenges in the genomic analysis of an endangered mammal population with low genetic diversity
Source: Sci Rep. 2022 Dec 10;12:21390. doi: 10.1038/s41598-022-25619-y (PMC9741620; doi:10.1038/s41598-022-25619-y)
Supplement: Supplementary file 1 — Supplementary Information. [file 41598_2022_25619_MOESM1_ESM.pdf]

**Supporting Information:** Tables and Figures

# Methodological challenges in the genomic analysis of an endangered mammal population with low genetic diversity

Lídia Escoda *[1], Oliver Hawlitschek *[1,2], Jorge González-Esteban [3], Jose Castresana [1]

[1] Institute of Evolutionary Biology (CSIC-Universitat Pompeu Fabra), Passeig Marítim de la Barceloneta 37, 08003 Barcelona, Spain

[2] Leibniz Institute for the Analysis of Biodiversity Change, Centre for Molecular Biodiversity Research, Zoological Museum, Martin-Luther-King-Platz 3, 20146 Hamburg, Germany

[3] Desma Estudios Ambientales S.L., Sunbilla (Navarra), Spain

* Joint first authors

Corresponding author: Jose Castresana
Institute of Evolutionary Biology (CSIC-Universitat Pompeu Fabra), Passeig Marítim de la Barceloneta 37, 08003 Barcelona, Spain

**Table S1.** Specimens used in this study together with information about their sex, collection year, and locality data. Specimens used in previous studies are indicated, as well as those used for the duplicate detection analysis.

| Specimen code | Sex | Collection year | Autonomous community | Locality | Locality code | Lat. | Long. | Duplicate analysis |
|---|---|---|---|---|---|---|---|---|
| IBE-C2755 [(1,2)] | Male | 2001 | Gipuzkoa | Leitzaran-Berastegi | 3 | 43.1 | -1.9 | |
| IBE-C2756 [(1,2)] | Female | 2001 | Gipuzkoa | Aiaiturrieta-Ataun | 1 | 43.0 | -2.1 | |
| IBE-C2757 [(1,2)] | Female | 2006 | Gipuzkoa | Amundarain-Zaldibia | 2 | 43.0 | -2.1 | |
| IBE-C2758 | Female | 2006 | Gipuzkoa | Amundarain-Zaldibia | 2 | 43.0 | -2.1 | X |
| IBE-C2759 | Male | 2001 | Gipuzkoa | Amundarain-Zaldibia | 2 | 43.0 | -2.1 | |
| IBE-C2760 [(1)] | Female | 2010 | Navarra | Erasote-Leitza | 4 | 43.1 | -1.9 | |
| IBE-C2761 | Female | 2010 | Navarra | Erasote-Leitza | 4 | 43.1 | -1.9 | X |
| IBE-C2762 | Male | 2010 | Navarra | Erasote-Leitza | 4 | 43.1 | -1.9 | X |
| IBE-C2763 [(1,2)] | Male | 2008 | Navarra | Olazar-Eugi | 10 | 43.0 | -1.5 | X |
| IBE-C2765 [(1,2)] | Male | 2010 | Navarra | Ezpelura-Urrotz | 8 | 43.1 | -1.7 | X |
| IBE-C2766 | Female | 1999 | Navarra | Ezpelura-Urrotz | 8 | 43.1 | -1.7 | X |
| IBE-C2769 [(3)] | Male | 1999 | Navarra | Ezpelura-Urrotz | 8 | 43.1 | -1.7 | |
| IBE-C2770 | Female | 1999 | Navarra | Ezpelura-Urrotz | 8 | 43.1 | -1.7 | X |
| IBE-C2771 | Male | 1999 | Navarra | Ezpelura-Urrotz | 8 | 43.1 | -1.7 | X |
| IBE-C2772 | Male | 1999 | Navarra | Ezpelura-Urrotz | 8 | 43.1 | -1.7 | X |
| IBE-C2773 | Male | 1999 | Navarra | Ezpelura-Urrotz | 8 | 43.1 | -1.7 | X |
| IBE-C2776 | Female | 1999 | Navarra | Ezpelura-Urrotz | 8 | 43.1 | -1.7 | X |
| IBE-C2778 | Male | 1999 | Navarra | Ezpelura-Urrotz | 8 | 43.1 | -1.7 | |
| IBE-C2779 | Female | 1999 | Navarra | Ezpelura-Urrotz | 8 | 43.1 | -1.7 | |
| IBE-C2780 | Male | 1999 | Navarra | Ezpelura-Urrotz | 8 | 43.1 | -1.7 | |
| IBE-C2781 [(1)] | Female | 1998 | Navarra | Ameztia-Labaien | 7 | 43.1 | -1.7 | X |
| IBE-C2782 | Female | 1998 | Navarra | Ameztia-Labaien | 7 | 43.1 | -1.7 | |
| IBE-C2783 | Female | 1998 | Navarra | Ameztia-Labaien | 7 | 43.1 | -1.7 | |
| IBE-C2784 | Male | 1998 | Navarra | Ameztia-Labaien | 7 | 43.1 | -1.7 | |
| IBE-C2785 | Female | 1998 | Navarra | Ameztia-Labaien | 7 | 43.1 | -1.7 | X |
| IBE-C2786 | Male | 1998 | Navarra | Ameztia-Labaien | 7 | 43.1 | -1.7 | |
| IBE-C2787 | Male | 1998 | Navarra | Ameztia-Labaien | 7 | 43.1 | -1.7 | |
| IBE-C2788 | Female | 1998 | Navarra | Ameztia-Labaien | 7 | 43.1 | -1.7 | |
| IBE-C2789 | Female | 1998 | Navarra | Ameztia-Labaien | 7 | 43.1 | -1.7 | |
| IBE-C2790 | Male | 1998 | Navarra | Ameztia-Labaien | 7 | 43.1 | -1.7 | X |
| IBE-C2791 [(1)] | Male | 2001 | Navarra | Urrobi-Auritz | 12 | 43.0 | -1.3 | X |
| IBE-C2792 | Female | 2001 | Navarra | Urrobi-Auritz | 12 | 43.0 | -1.3 | |
| IBE-C2793 | Female | 2001 | Navarra | Urrobi-Auritz | 12 | 43.0 | -1.3 | X |
| IBE-C2794 | Male | 2001 | Navarra | Urrobi-Auritz | 12 | 43.0 | -1.3 | X |
| IBE-C2796 [(1)] | Male | 1997 | Navarra | Sasoaran-Eugi | 9 | 43.0 | -1.6 | X |
| IBE-C2797 | Female | 1997 | Navarra | Sasoaran-Eugi | 9 | 43.0 | -1.6 | X |
| IBE-C2799 [(1,2)] | Male | 2002 | Navarra | Elama-Artikutza | 6 | 43.2 | -1.8 | X |
| IBE-C2800 | Male | 2002 | Navarra | Elama-Artikutza | 6 | 43.2 | -1.8 | |
| IBE-C2802 | Female | 2002 | Navarra | Elama-Artikutza | 6 | 43.2 | -1.8 | X |
| IBE-C2803 | Male | 2002 | Navarra | Elama-Artikutza | 6 | 43.2 | -1.8 | X |
| IBE-C2804 | Male | 2002 | Navarra | Elama-Artikutza | 6 | 43.2 | -1.8 | |
| IBE-C3759 [(1)] | Female | 2011 | Navarra | Urumea | 5 | 43.1 | -1.8 | |
| IBE-C3760 | Male | 2011 | Navarra | Urumea | 5 | 43.1 | -1.8 | X |
| IBE-C3761 | Female | 2011 | Navarra | Urumea | 5 | 43.1 | -1.8 | |
| IBE-C3763 [(1)] | Male | 2011 | Navarra | Aritzakun | 11 | 43.3 | -1.4 | |

[(1)] Igea et al. 2013
[(2)] Querejeta et al. 2016
[(3)] Escoda & Castresana 2021

**Table S2.** Basic statistics of the library sequences.

| Specimen code | Raw reads | Mapped reads | Endogenous DNA (%) | Assembled reads | Assembled loci | Coverage |
|---|---|---|---|---|---|---|
| IBE-C2755 | 2,055,750 | 1,938,994 | 94.0 | 1,870,836 | 67,986 | 27.52 |
| IBE-C2756 | 3,223,077 | 3,018,212 | 92.1 | 2,882,597 | 72,135 | 39.96 |
| IBE-C2757 | 2,263,306 | 2,013,822 | 87.4 | 1,922,067 | 77,249 | 24.88 |
| IBE-C2758 | 2,458,337 | 2,328,368 | 92.7 | 2,216,933 | 89,676 | 24.72 |
| IBE-C2759 | 4,980,296 | 4,614,458 | 95.2 | 4,372,024 | 106,777 | 40.95 |
| IBE-C2760 | 2,960,005 | 2,825,996 | 95.3 | 2,756,340 | 83,828 | 32.88 |
| IBE-C2761 | 5,961,091 | 5,598,014 | 93.2 | 5,364,690 | 245,676 | 21.84 |
| IBE-C2762 | 8,093,377 | 7,690,778 | 93.3 | 7,296,115 | 128,845 | 56.63 |
| IBE-C2763 | 3,002,097 | 2,830,037 | 92.8 | 2,698,121 | 113,114 | 23.85 |
| IBE-C2765 | 1,026,994 | 938,665 | 92.2 | 889,224 | 123,086 | 7.22 |
| IBE-C2766 | 4,224,186 | 4,006,009 | 94.3 | 3,861,489 | 144,912 | 26.65 |
| IBE-C2769 | 7,450,879 | 7,028,134 | 97.0 | 6,868,555 | 140,827 | 48.77 |
| IBE-C2770 | 6,297,156 | 5,925,101 | 94.2 | 5,725,200 | 108,466 | 52.78 |
| IBE-C2771 | 5,511,207 | 5,175,133 | 93.3 | 4,961,777 | 90,260 | 54.97 |
| IBE-C2772 | 2,675,478 | 2,529,177 | 91.3 | 2,390,907 | 93,071 | 25.69 |
| IBE-C2773 | 2,706,390 | 2,578,524 | 94.8 | 2,486,606 | 108,980 | 22.82 |
| IBE-C2776 | 3,744,171 | 3,542,745 | 94.3 | 3,439,677 | 103,272 | 33.31 |
| IBE-C2778 | 3,315,499 | 3,125,425 | 93.3 | 3,010,148 | 73,115 | 41.17 |
| IBE-C2779 | 2,884,397 | 2,719,137 | 93.8 | 2,618,920 | 68,756 | 38.09 |
| IBE-C2780 | 1,477,552 | 1,390,509 | 94.0 | 1,346,293 | 62,688 | 21.48 |
| IBE-C2781 | 3,343,999 | 3,168,191 | 94.0 | 3,033,611 | 125,513 | 24.17 |
| IBE-C2782 | 2,289,454 | 2,174,068 | 94.7 | 2,102,724 | 69,885 | 30.09 |
| IBE-C2783 | 4,655,418 | 4,382,020 | 94.2 | 4,209,765 | 101,745 | 41.38 |
| IBE-C2784 | 2,332,204 | 2,201,928 | 94.8 | 2,124,863 | 106,011 | 20.04 |
| IBE-C2785 | 5,662,179 | 5,410,028 | 95.5 | 5,253,044 | 134,903 | 38.94 |
| IBE-C2786 | 2,803,468 | 2,650,241 | 92.6 | 2,537,459 | 70,643 | 35.92 |
| IBE-C2787 | 9,257,109 | 8,721,343 | 92.4 | 8,347,773 | 96,398 | 86.60 |
| IBE-C2788 | 8,677,240 | 8,155,052 | 94.4 | 7,995,427 | 91,428 | 87.45 |
| IBE-C2789 | 14,451,258 | 13,528,925 | 93.4 | 13,020,104 | 126,058 | 103.29 |
| IBE-C2790 | 5,543,162 | 5,290,531 | 93.4 | 5,045,665 | 121,054 | 41.68 |
| IBE-C2791 | 3,912,245 | 3,714,414 | 94.3 | 3,583,827 | 119,017 | 30.11 |
| IBE-C2792 | 5,914,177 | 5,583,584 | 94.4 | 5,464,216 | 92,701 | 58.95 |
| IBE-C2793 | 6,226,362 | 5,843,557 | 94.4 | 5,589,272 | 142,095 | 39.34 |
| IBE-C2794 | 1,239,874 | 1,165,090 | 90.1 | 1,089,342 | 84,955 | 12.82 |
| IBE-C2796 | 3,263,897 | 3,058,690 | 93.0 | 2,908,275 | 136,505 | 21.31 |
| IBE-C2797 | 2,395,890 | 2,249,648 | 92.3 | 2,143,303 | 123,396 | 17.37 |
| IBE-C2799 | 1,982,105 | 1,868,503 | 92.8 | 1,791,686 | 87,774 | 20.41 |
| IBE-C2800 | 1,307,909 | 1,206,538 | 91.4 | 1,163,500 | 77,659 | 14.98 |
| IBE-C2802 | 2,267,684 | 2,090,032 | 92.4 | 2,010,656 | 128,740 | 15.62 |
| IBE-C2803 | 3,033,378 | 2,830,375 | 92.5 | 2,731,835 | 95,752 | 28.53 |
| IBE-C2804 | 4,668,208 | 4,423,466 | 93.8 | 4,282,172 | 79,822 | 53.65 |
| IBE-C3759 | 2,183,830 | 2,002,690 | 91.2 | 1,948,089 | 77,866 | 25.02 |
| IBE-C3760 | 6,340,281 | 5,997,205 | 94.1 | 5,741,970 | 123,818 | 46.37 |
| IBE-C3761 | 3,608,024 | 3,406,431 | 93.8 | 3,305,462 | 83,814 | 39.44 |
| IBE-C3763 | 5,116,317 | 4,856,410 | 93.2 | 4,669,466 | 84,757 | 55.09 |

**Table S3.** Individual inbreeding coefficients and heterozygosity of the specimens.

| Specimen code | Locality | Locality code | Inbreeding coefficient (RELATED) | Inbreeding coefficient (PLINK) | Heterozygosity (SNPs/Mb) |
|---|---|---|---|---|---|
| IBE-C2755 | Leitzaran-Berastegi | 3 | 0.2834 | 0.2202 | 74 |
| IBE-C2756 | Aiaiturrieta-Ataun | 1 | 0.6941 | 0.6418 | 37 |
| IBE-C2757 | Amundarain-Zaldibia | 2 | 0.7904 | 0.7489 | 26 |
| IBE-C2758 | Amundarain-Zaldibia | 2 | 0.7812 | 0.7383 | 26 |
| IBE-C2759 | Amundarain-Zaldibia | 2 | 0.7576 | 0.7127 | 30 |
| IBE-C2760 | Erasote-Leitza | 4 | 0.3801 | 0.3236 | 69 |
| IBE-C2761 | Erasote-Leitza | 4 | 0.3024 | 0.2380 | 75 |
| IBE-C2762 | Erasote-Leitza | 4 | 0.3761 | 0.3290 | 63 |
| IBE-C2763 | Olazar-Eugi | 10 | 0.2425 | 0.1483 | 88 |
| IBE-C2765 | Ezpelura-Urrotz | 8 | 0.1397 | 0.2039 | 71 |
| IBE-C2766 | Ezpelura-Urrotz | 8 | 0.1353 | 0.2014 | 77 |
| IBE-C2769 | Ezpelura-Urrotz | 8 | 0.1052 | 0.1834 | 80 |
| IBE-C2770 | Ezpelura-Urrotz | 8 | 0.0175 | 0.1070 | 82 |
| IBE-C2771 | Ezpelura-Urrotz | 8 | 0.0410 | 0.0801 | 85 |
| IBE-C2772 | Ezpelura-Urrotz | 8 | 0.1390 | 0.1959 | 79 |
| IBE-C2773 | Ezpelura-Urrotz | 8 | 0.0528 | 0.1044 | 87 |
| IBE-C2776 | Ezpelura-Urrotz | 8 | 0.0574 | 0.1195 | 82 |
| IBE-C2778 | Ezpelura-Urrotz | 8 | 0.1307 | 0.1898 | 75 |
| IBE-C2779 | Ezpelura-Urrotz | 8 | 0.0573 | 0.1231 | 84 |
| IBE-C2780 | Ezpelura-Urrotz | 8 | 0.1556 | 0.2529 | 73 |
| IBE-C2781 | Ameztia-Labaien | 7 | 0.0375 | 0.0804 | 89 |
| IBE-C2782 | Ameztia-Labaien | 7 | 0.2495 | 0.3038 | 70 |
| IBE-C2783 | Ameztia-Labaien | 7 | 0.0980 | 0.1722 | 77 |
| IBE-C2784 | Ameztia-Labaien | 7 | 0.1217 | 0.1831 | 74 |
| IBE-C2785 | Ameztia-Labaien | 7 | 0.0836 | 0.1296 | 80 |
| IBE-C2786 | Ameztia-Labaien | 7 | 0.1416 | 0.1555 | 80 |
| IBE-C2787 | Ameztia-Labaien | 7 | 0.0144 | 0.0417 | 91 |
| IBE-C2788 | Ameztia-Labaien | 7 | 0.0876 | 0.1602 | 77 |
| IBE-C2789 | Ameztia-Labaien | 7 | 0.0738 | 0.1133 | 80 |
| IBE-C2790 | Ameztia-Labaien | 7 | 0.3772 | 0.4019 | 57 |
| IBE-C2791 | Urrobi-Auritz | 12 | 0.3439 | 0.2814 | 72 |
| IBE-C2792 | Urrobi-Auritz | 12 | 0.3791 | 0.3244 | 65 |
| IBE-C2793 | Urrobi-Auritz | 12 | 0.3740 | 0.3288 | 67 |
| IBE-C2794 | Urrobi-Auritz | 12 | 0.3963 | 0.3550 | 61 |
| IBE-C2796 | Sasoaran-Eugi | 9 | 0.3669 | 0.3110 | 71 |
| IBE-C2797 | Sasoaran-Eugi | 9 | 0.3719 | 0.3385 | 63 |
| IBE-C2799 | Elama-Artikutza | 6 | 0.2006 | 0.1229 | 83 |
| IBE-C2800 | Elama-Artikutza | 6 | 0.4783 | 0.4680 | 55 |
| IBE-C2802 | Elama-Artikutza | 6 | 0.2257 | 0.1979 | 78 |
| IBE-C2803 | Elama-Artikutza | 6 | 0.2447 | 0.2307 | 73 |
| IBE-C2804 | Elama-Artikutza | 6 | 0.2770 | 0.2512 | 72 |
| IBE-C3759 | Urumea | 5 | 0.1982 | 0.1542 | 85 |
| IBE-C3760 | Urumea | 5 | 0.1687 | 0.0619 | 89 |
| IBE-C3761 | Urumea | 5 | 0.1313 | 0.0481 | 90 |
| IBE-C3763 | Aritzakun | 11 | 0.2851 | 0.2410 | 77 |

**Tables S4**. Results of the individual identification performed with different programs and with different values of the MAF filter, indicating the number of SNPs generated for each dataset. Correct detection of replicas corresponds to the detection of the 23 pairs of duplicated samples. Correct detection of highly inbred individuals corresponds to the detection of the 4 available sequencing experiments of the 3 individuals from Amundarain-Zaldibia.

| | **MAF = 0.1 (479 SNPs)** | |
|---|---|---|
| **Program** | **Correct detection of replicas** | **Correct detection of highly inbred individuals** |
| RELATED | 23/23 | 0/5 |
| KING | 23/23 | 5/5 |
| KING --*duplicate* | 21/23 | 5/5 |
| PLINK | 23/23 | 0/5 |
| COLONY | 23/23 | 0/5 |
| PRIMUS | 23/23 | 0/5 |
| VCF2LR | 23/23 | 0/5 |

| | **MAF = 0.2 (328 SNPs)** | |
|---|---|---|
| **Program** | **Correct detection of replicas** | **Correct detection of highly inbred individuals** |
| RELATED | 23/23 | 0/5 |
| KING | 23/23 | 5/5 |
| KING --*duplicate* | 21/23 | 5/5 |
| PLINK | 23/23 | 0/5 |
| COLONY | 23/23 | 0/5 |
| PRIMUS | 23/23 | 0/5 |
| VCF2LR | 23/23 | 0/5 |

| | **MAF = 0.3 (199 SNPs)** | |
|---|---|---|
| **Program** | **Correct detection of replicas** | **Correct detection of highly inbred individuals** |
| RELATED | 23/23 | 0/5 |
| KING | 23/23 | 5/5 |
| KING --*duplicate* | 22/23 | 4/5 |
| PLINK | 23/23 | 0/5 |
| COLONY | 23/23 | 0/5 |
| PRIMUS | 23/23 | 0/5 |
| VCF2LR | 23/23 | 0/5 |

**Table S5**. Relatedness estimated with simulated pedigrees using different reference populations and the program RELATED. Means and standard deviations (in parentheses) are given.

| Relationship | Theoretical value | Observed Value (SD) | | |
|---|---|---|---|---|
| | | Western area | Central area | Eastern area |
| Parent-offspring | 0.5 | 0.6006 (0.0592) | 0.5620 (0.0544) | 0.6633 (0.0654) |
| Full-siblings | 0.5 | 0.6005 (0.0187) | 0.5369 (0.0255) | 0.6866 (0.0260) |
| Half-siblings | 0.25 | 0.3197 (0.0226) | 0.3008 (0.0287) | 0.3456 (0.0214) |
| Grandparent-grandchild | 0.25 | 0.3015 (0.0247) | 0.3169 (0.0402) | 0.3727 (0.0395) |
| Uncle-nephew | 0.25 | 0.3061 (0.0260) | 0.3013 (0.0351) | 0.3636 (0.0274) |
| Half uncle-half nephew | 0.125 | 0.2283 (0.0308) | 0.2067 (0.0443) | 0.3726 (0.0513) |
| Half-first cousins | 0.0625 | 0.1784 (0.0302) | 0.2061 (0.0335) | 0.2883 (0.0230) |

**Table S6**. Relatedness estimated with simulated pedigrees using different reference populations and the program KING, where the kinship coefficients obtained have been doubled to be comparable to the relatedness coefficients obtained with RELATED. Means and standard deviations (in parentheses) are given.

| **Relationship** | **Theoretical value** | **Observed Value (SD)** | | |
|---|---|---|---|---|
| | | **Western area** | **Central area** | **Eastern area** |
| Parent-offspring | 0.5 | 0.4505 (0.0481) | 0.4974 (0.0326) | 0.4428 (0.0330) |
| Full-siblings | 0.5 | 0.6005 (0.0266) | 0.4888 (0.0317) | 0.6203 (0.0258) |
| Half-siblings | 0.25 | 0.3374 (0.0288) | 0.2528 (0.0393) | 0.2618 (0.0305) |
| Grandparent-grandchild | 0.25 | 0.1081 (0.0783) | 0.2602 (0.0600) | 0.0742 (0.0994) |
| Uncle-nephew | 0.25 | 0.3102 (0.0346) | 0.2456 (0.0448) | 0.3024 (0.0393) |
| Half uncle-half nephew | 0.125 | 0.2341 (0.0587) | 0.1543 (0.0612) | 0.3122 (0.0374) |
| Half-first cousins | 0.0625 | 0.1691 (0.0435) | 0.1354 (0.0488) | 0.2421 (0.0369) |

**Table S7**. Individual inbreeding coefficients and standard deviations (in parentheses) estimated with simulated pedigrees of offspring (in parentheses) from different types of parental relationships and using different referent populations. Offspring codes can be found in supplementary Figure S2.

| **Parental relationship (offspring)** | **Theoretical value** | **Observed Value (SD)** | | |
|---|---|---|---|---|
| | | **Western area** | **Central area** | **Eastern area** |
| None (F1, F201, F202) | 0 | 0.0033 (0.0079) | 0.0105 (0.0168) | 0.0159 (0.0175) |
| Full-siblings (F203, F205) | 0.25 | 0.2610 (0.0289) | 0.2433 (0.0350) | 0.3244 (0.0295) |
| Half-siblings (F204) | 0.125 | 0.1250 (0.0297) | 0.1159 (0.0327) | 0.1382 (0.0350) |
| Half-first cousins (F301) | 0.03125 | 0.0522 (0.0276) | 0.0792 (0.0296) | 0.1118 (0.0312) |

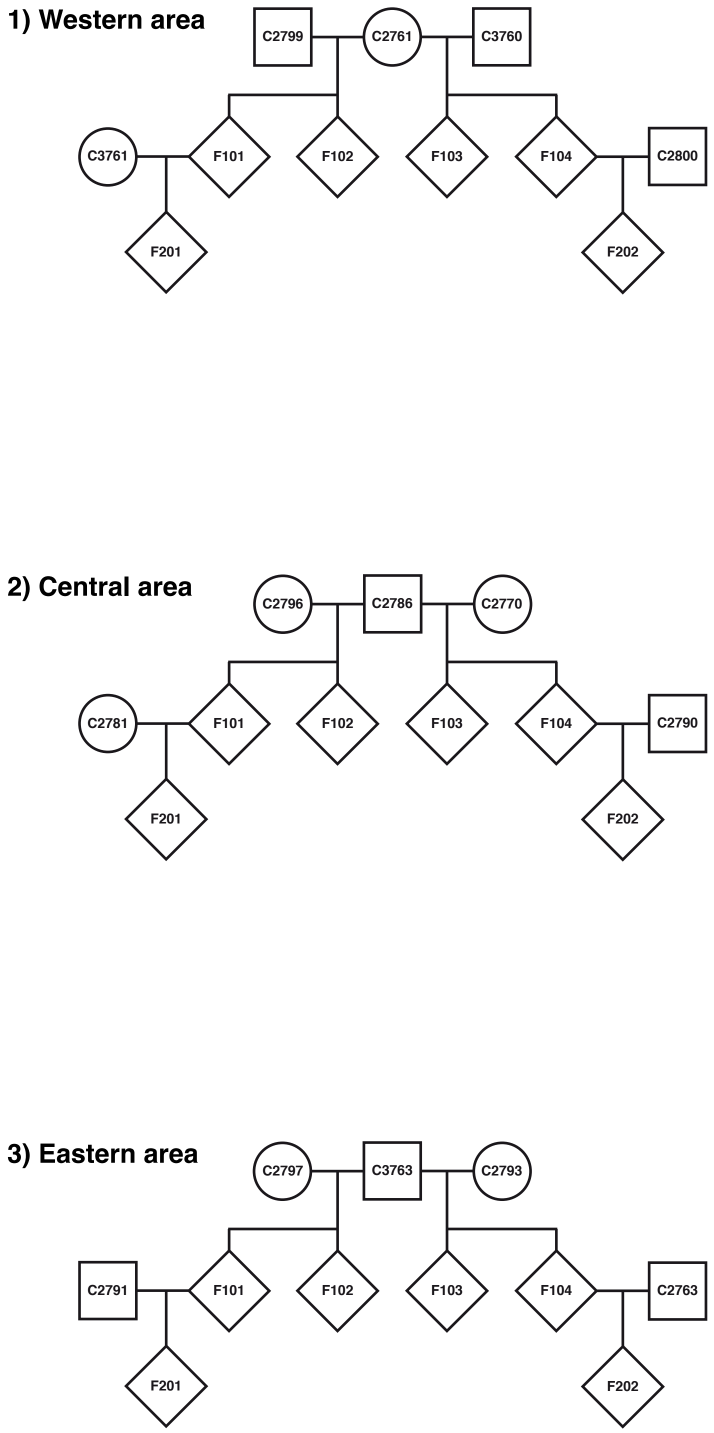


**Figure S1.** Pedigrees used for simulations of relatedness between individuals. Founder males are represented with squares, founder females with circles and simulated offspring with diamonds. The identity of the founders can be found in Table S1. Founders were chosen by areas: pedigree 1 corresponds to individuals in the occidental area (localities 3-6), pedigree 2 to the central area (localities 7-8), and pedigree 3 to the eastern area (localities 9-12).

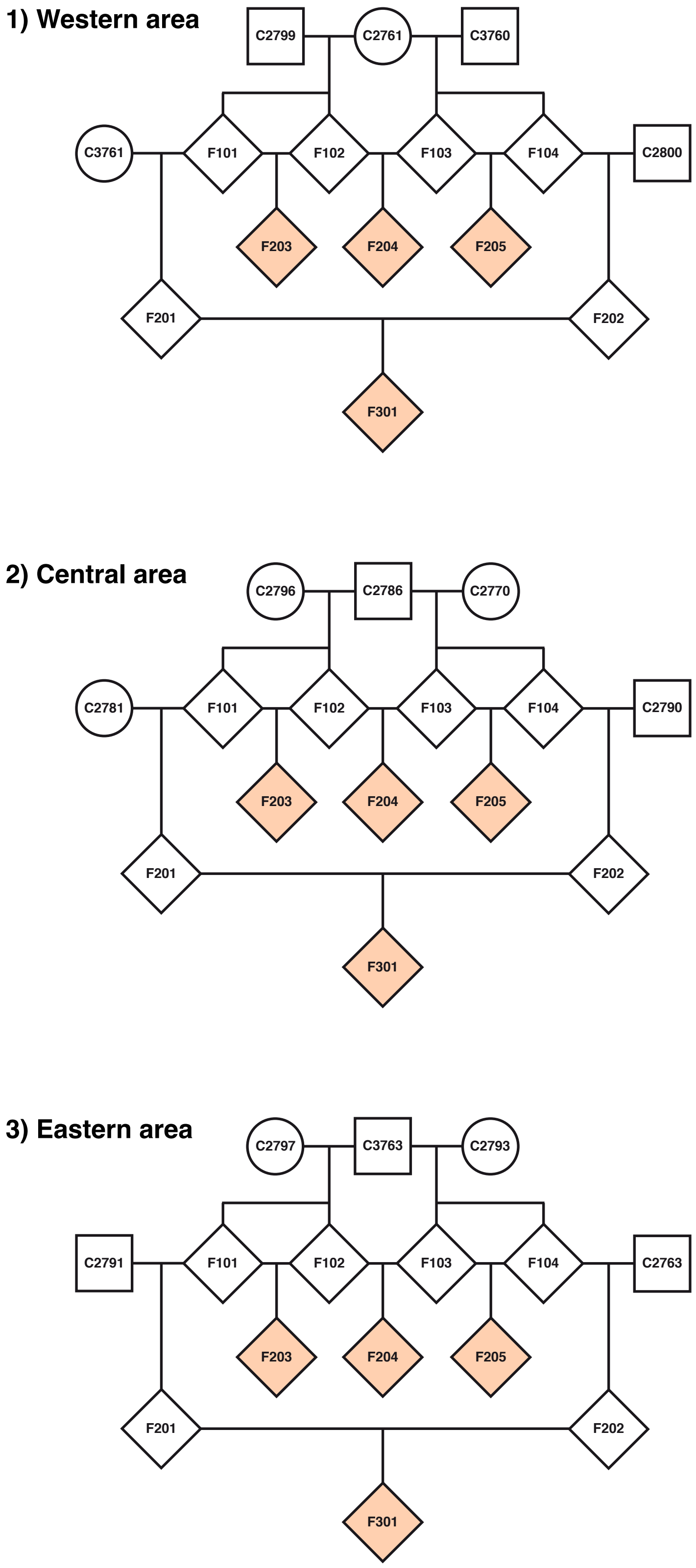


**Figure S2.** Pedigrees used for simulations of individual inbreeding coefficients. Founder males are represented with squares, founder females with circles and simulated offspring with diamonds. Inbred individuals are represented in light red. The identity of the founders can be found in Table S1. Founders were chosen by areas: pedigree 1 corresponds to individuals in the occidental area (localities 3-6), pedigree 2 to the central area (localities 7-8), and pedigree 3 to the eastern area (localities 9-12).

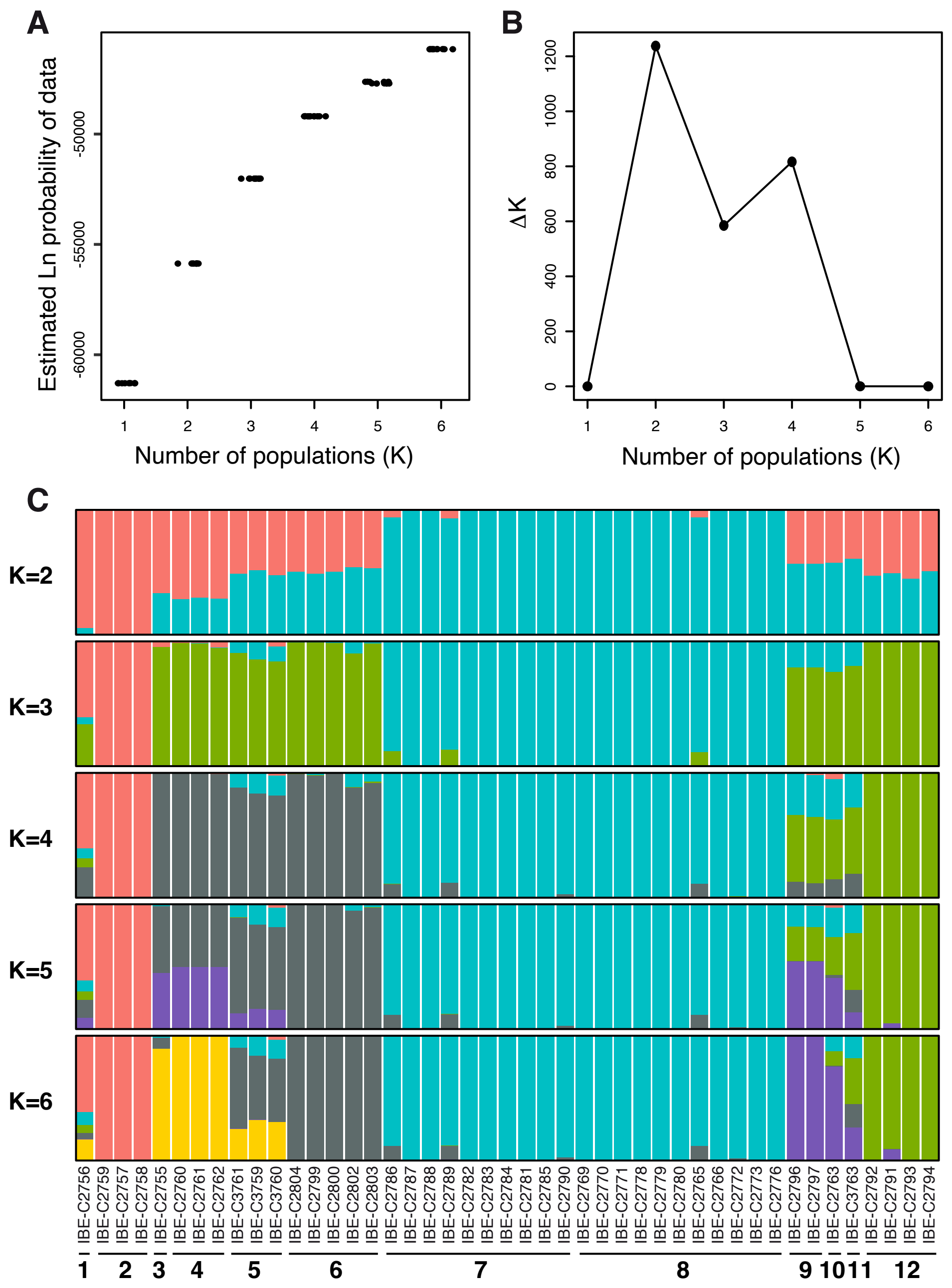


**Figure S3.** Population structure analysis of the individuals. (A) Log likelihood of data and (B) delta graph of the STRUCTURE analysis according to the method from Evanno et al. (2005). (C) Barplots of admixture proportions of each specimen for different number of populations (K). Specimen code and locality code are indicated for each individual as in Table S1.

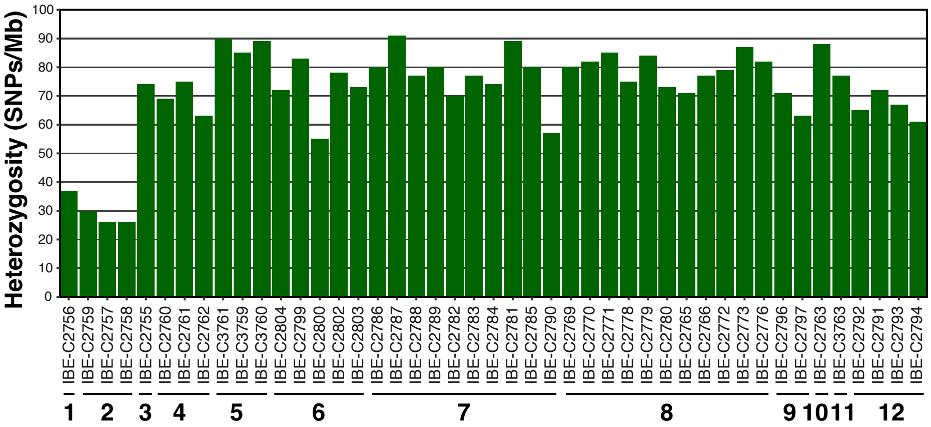


**Figure S4.** Heterozygosity of Iberian desmans in SNPs/Mb sorted by geographic longitude.

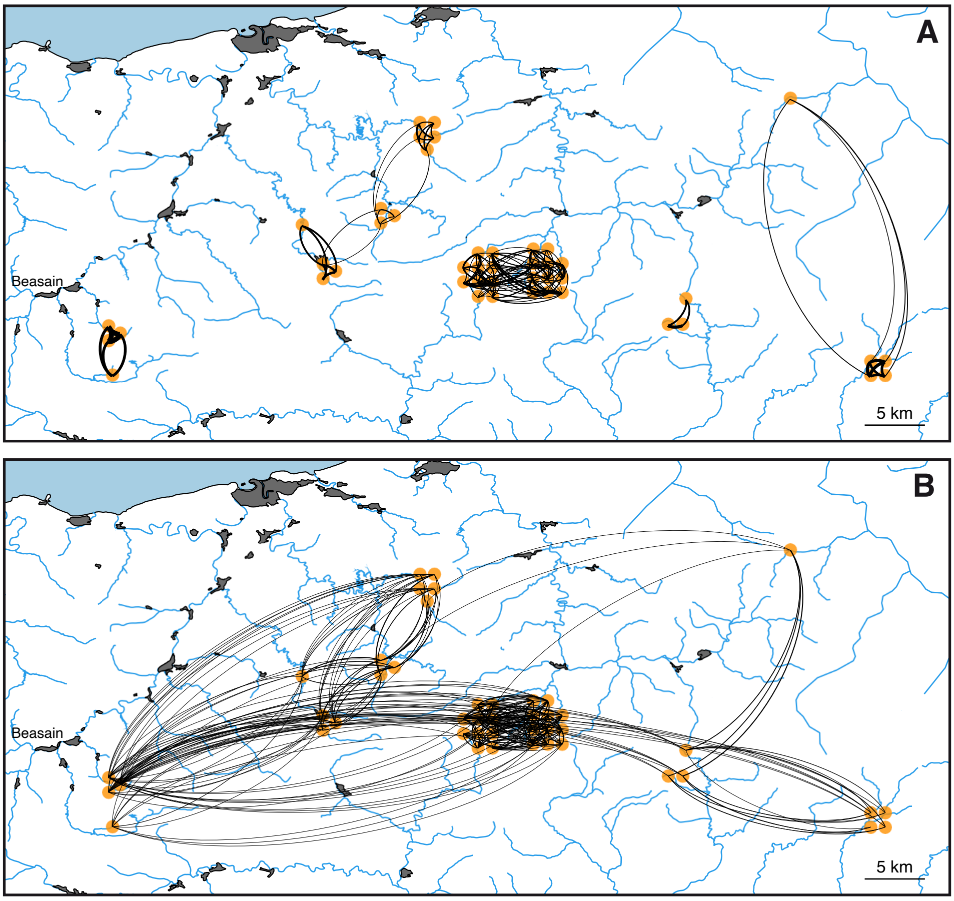


**Figure S5.** Maps plotting networks of related individuals estimated with RELATED. (A) Close relationships with a relatedness coefficient above 0.2 and (B) distant relationships with a relatedness coefficient below 0.2. Each line thickness is proportional to the relatedness coefficient of the connected Iberian desmans. The map was constructed as indicated in the legend to figure 1.

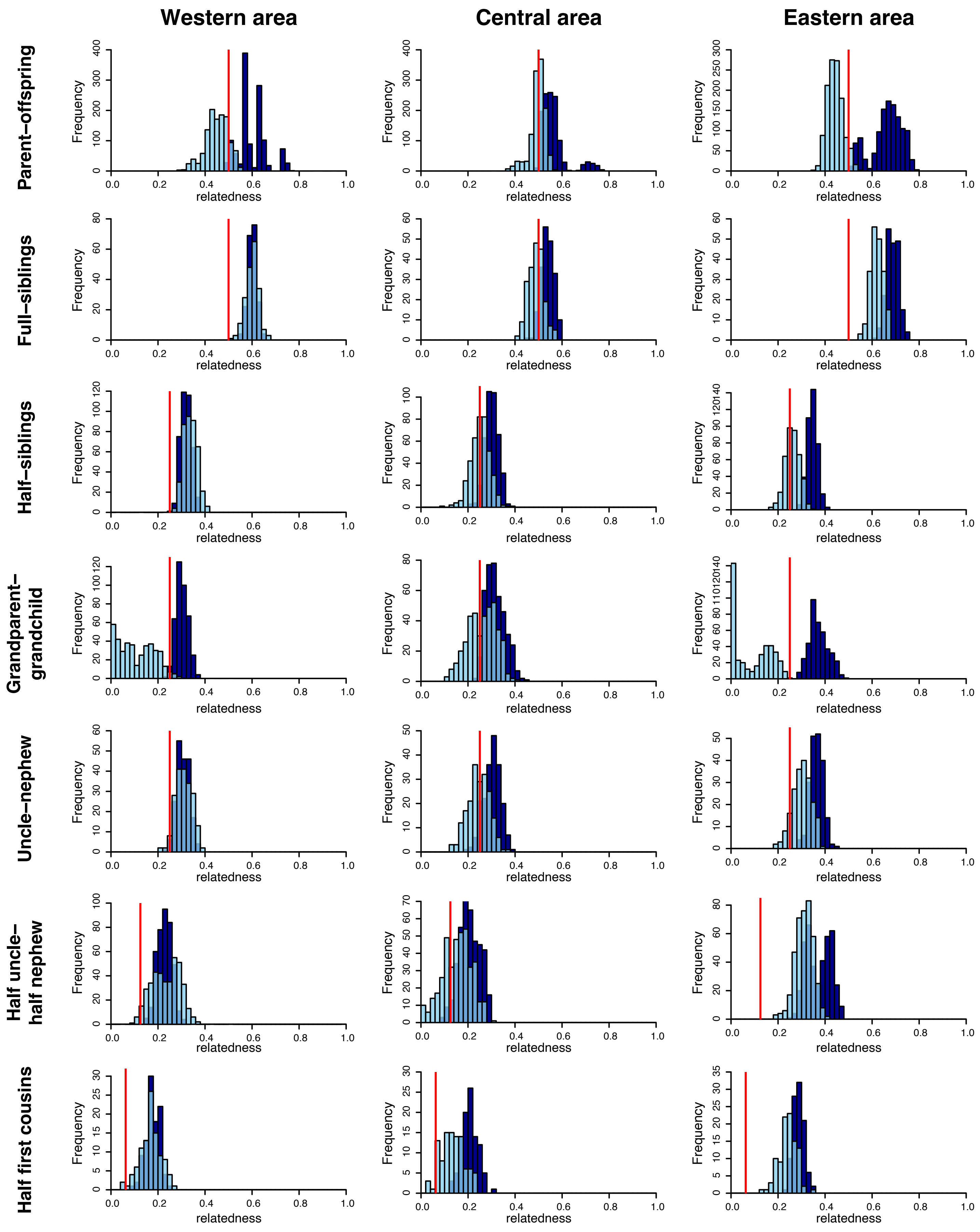


**Figure S6.** Frequency histograms of the relatedness values obtained from the simulations performed along artificial pedigrees with the programs RELATED (dark blue) and KING (light blue). Red lines indicate the expected values for each category.

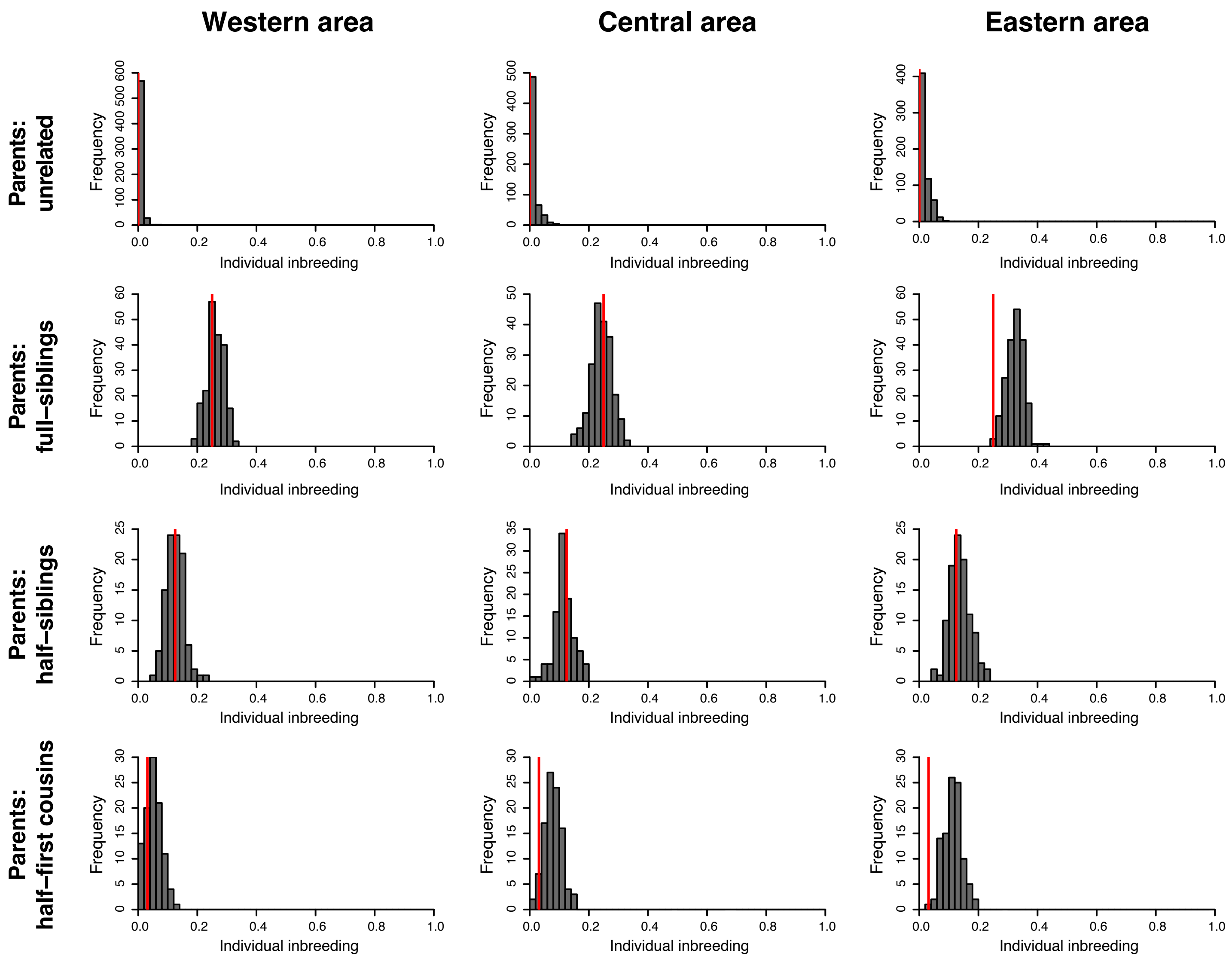


**Figure S7.** Frequency histograms of the individual inbreeding coefficients obtained from the simulations performed along artificial pedigrees with crosses of pairs with known parental relationships. Red lines indicate the expected values for each category.
